# Supplementary material for: CRISPR-enabled point-of-care genotyping for APOL1 genetic risk assessment
Source: EMBO Mol Med. 2024 Sep 13;16(10):18. doi: 10.1038/s44321-024-00126-x (PMC11473833; doi:10.1038/s44321-024-00126-x)
Supplement: Supplementary file 1 — Appendix [file 44321_2024_126_MOESM1_ESM.pdf]

Appendix

CRISPR-Enabled Point-of-Care Genotyping for *APOL1* Genetic Risk Assessment

Robert Greensmith[1,2], Isadora T. Lape[3], Cristian V. Riella[4,5], Alexander J. Schubert[1,2,6], Jakob Metzger[1], Anand S. Dighe[5,7], Xiao Tan[8,9,10,11], Bernhard Hemmer[12,13], Josefine Rau[1], Sarah Wendlinger[1,2], Nora Diederich[1,2], Anja Schütz[14], Leonardo V. Riella[3,15,16]*, Michael M. Kaminski[1,2,6]*

[1] Berlin Institute for Medical Systems Biology, Max Delbrück Center for Molecular Medicine in the Helmholtz Association, Berlin, Germany.
[2] Department of Nephrology and Medical Intensive Care, Charité Universitätsmedizin Berlin, Berlin, Germany.
[3] Center for Transplantation Sciences, Massachusetts General Hospital/Harvard Medical School, Boston, MA, United States.
[4] Nephrology Division, Department of Medicine, Beth Israel Deaconess Medical Center, Boston, MA 02215.
[5] Harvard Medical School, Boston, MA 02115.
[6] Berlin Institute of Health, Berlin, Germany.
[7] Department of Pathology, Massachusetts General Hospital, Boston, MA, USA.
[8] Wyss Institute for Biologically Inspired Engineering, Harvard University, Boston, MA, USA.
[9] Division of Gastroenterology, Massachusetts General Hospital, Boston, MA, USA.
[10] Institute for Medical Engineering and Science and Department of Biological Engineering, Massachusetts Institute of Technology, MA, USA.
[11] Infectious Disease and Microbiome Program, Broad Institute of MIT and Harvard, Cambridge, MA, USA.
[12] Department of Neurology, Klinikum rechts der Isar, Technical University of Munich, Munich, Germany.
[13] Munich Cluster for Systems Neurology (SyNergy), Munich, Germany.
[14] Protein Production & Characterization, Max Delbrück Center for Molecular Medicine in the Helmholtz Association, 13125, Berlin, Germany.
[15] Department of Surgery, Massachusetts General Hospital/Harvard Medical School, Boston, MA, United States.
[16] Division of Nephrology, Massachusetts General Hospital/Harvard Medical School, Boston, MA, United States.

***Corresponding authors. Emails:** lriella@mgh.harvard.edu; michael.kaminski@mdc-berlin.de

Table of Contents

Appendix Table S1 2
Appendix Table S2 9
Appendix Table S3 13

Appendix Table S1: Oligonucleotides included in this study.

Description	Used in	Sequence (5' - 3')
APOL1 G1 wildtype; Construct for *Off-target (A)*	Fig. 1b	gaaattaatacgactcactataggGCCAATCTCAGCTGAAAGCGGTGAACAGGTGGAGAGGGTTAATGAACCCAGCATCCTGGAAATGAGCAGAGGAGTCAAGCTCACGGATGTGGCCCCTGTAAGCTTCTTTCTTGTGCTGGATGTAGTCTACCTCGTGTACGAATCAAAGCACTTACATGAGGGGGCAAAGTCAGAGACAGCTGAGGAGCTGAAGAAGGTGGC
APOL1 G1 mutant; Construct for *On-target (G)*	Figs. 1b, 2a	gaaattaatacgactcactataggGCCAATCTCAGCTGAAAGCGGTGAACAGGTGGAGAGGGTTAATGAACCCAGCATCCTGGAAATGAGCAGAGGAGTCAAGCTCACGGATGTGGCCCCTGTAGGCTTCTTTCTTGTGCTGGATGTAGTCTACCTCGTGTACGAATCAAAGCACTTACATGAGGGGGCAAAGTCAGAGACAGCTGAGGAGCTGAAGAAGGTGGC
Construct for crRNA1	Fig. 1b	CAAGCTCACGGATGTGGCCCCTGTGGGCGTTTTAGTCCCCTTCGTTTTTGGGGTAGTCTAAATCcctatagtgagtcgtattaatttc
Construct for crRNA2	Fig. 1b	CAAGCTCACGGATGTGGCCCCTGTAGGAGTTTTAGTCCCCTTCGTTTTTGGGGTAGTCTAAATCcctatagtgagtcgtattaatttc
Construct for crRNA3	Fig. 1b	CAAGCTCACGGATGTGGCCCCTGTAGGGGTTTTAGTCCCCTTCGTTTTTGGGGTAGTCTAAATCcctatagtgagtcgtattaatttc
Construct for crRNA4	Fig. 1b	CAAGCTCACGGATGTGGCCCCTGTAGGTGTTTTAGTCCCCTTCGTTTTTGGGGTAGTCTAAATCcctatagtgagtcgtattaatttc
Construct for crRNA5	Fig. 1b	CAAGCTCACGGATGTGGCCCCTGTGGGGGTTTTAGTCCCCTTCGTTTTTGGGGTAGTCTAAATCcctatagtgagtcgtattaatttc
Construct for crRNA6	Fig. 1b	CAAGCTCACGGATGTGGCCCCTGTGGCCGTTTTAGTCCCCTTCGTTTTTGGGGTAGTCTAAATCcctatagtgagtcgtattaatttc
Construct for crRNA7	Fig. 1b	CAAGCTCACGGATGTGGCCCCTGAGGGCGTTTTAGTCCCCTTCGTTTTTGGGGTAGTCTAAATCcctatagtgagtcgtattaatttc
Construct forcrRNA8	Fig. 1b	CAAGCTCACGGATGTGGCCCCTCTGGGCGTTTTAGTCCCCTTCGTTTTTGGGGTAGTCTAAATCcctatagtgagtcgtattaatttc
Construct for crRNA9	Fig. 1b	CAAGCTCACGGATGTGGCCCCAGTGGGCGTTTTAGTCCCCTTCGTTTTTGGGGTAGTCTAAATCcctatagtgagtcgtattaatttc

APOL1 G2 mutant; Construct for *Off-target (delTTATAA)*	Figs. 1d, EV3d	gaaattaatacgactcactataggATCAAAGCACTTACATGAGGGGGCAAAGTCAGAGACAGCTGAGGAGCTGAAGAAGGTGGCTCAGGAGCTGGAGGAGAAGCTAAACATTCTCAACAATAAGATTCTGCAGGCGGACCAAGAACTGTGACCACAGGGCAGGGCAGCCACCAGGAGAGATATGCCTGGCAGGGGCCAGGACAAAATGCAAACTT
APOL1 G2 wildtype; Construct for *On-target (TTATAA)*	Figs. 1d, 2d, EV3d	gaaattaatacgactcactataggATCAAAGCACTTACATGAGGGGGCAAAGTCAGAGACAGCTGAGGAGCTGAAGAAGGTGGCTCAGGAGCTGGAGGAGAAGCTAAACATTCTCAACAATAATTATAAGATTCTGCAGGCGGACCAAGAACTGTGACCACAGGGCAGGGCAGCCACCAGGAGAGATATGCCTGGCAGGGGCCAGGACAAAATGCAAACTT
Construct for crRNA10	Fig. 1d	GTTGTCATACCTATCCAAACGATAAGCTTCTACAACGAGGAGAAGCTAAACATTCTCAACAATAATcctatagtgagtcgtattaatttc
Construct for crRNA11	Fig. 1d	GTTGTCATACCTATCCAAACGATAAGCTTCTACAACAGGAGAAGCTAAACATTCTCAACAATAATTcctatagtgagtcgtattaatttc
Construct for crRNA12	Fig. 1d	GTTGTCATACCTATCCAAACGATAAGCTTCTACAACGGAGAAGCTAAACATTCTCAACAATAATTAcctatagtgagtcgtattaatttc
Construct for crRNA13	Fig. 1d	GTTGTCATACCTATCCAAACGATAAGCTTCTACAACGAGAAGCTAAACATTCTCAACAATAATTATcctatagtgagtcgtattaatttc
Construct for crRNA14	Fig. 1d	GTTGTCATACCTATCCAAACGATAAGCTTCTACAACAGAAGCTAAACATTCTCAACAATAATTATAcctatagtgagtcgtattaatttc
Construct for crRNA15	Fig. 1d	GTTGTCATACCTATCCAAACGATAAGCTTCTACAACGAAGCTAAACATTCTCAACAATAATTATAAcctatagtgagtcgtattaatttc
Construct for crRNA16	Fig. 1d	GTTGTCATACCTATCCAAACGATAAGCTTCTACAACAAGCTAAACATTCTCAACAATAATTATAAGcctatagtgagtcgtattaatttc
Construct for crRNA17	Fig. 1d	GTTGTCATACCTATCCAAACGATAAGCTTCTACAACAGCTAAACATTCTCAACAATAATTATAAGAcctatagtgagtcgtattaatttc
Construct for crRNA18	Fig. 1d	GTTGTCATACCTATCCAAACGATAAGCTTCTACAACGCTAAACATTCTCAACAATAATTATAAGATcctatagtgagtcgtattaatttc
Construct for crRNA19	Fig. 1d	GTTGTCATACCTATCCAAACGATAAGCTTCTACAACCTAAACATTCTCAACAATAATTATAAGATTcctatagtgagtcgtattaatttc
Construct for crRNA20	Fig. 1d	GTTGTCATACCTATCCAAACGATAAGCTTCTACAACTAAACATTCTCAACAATAATTATAAGATTCcctatagtgagtcgtattaatttc

Construct for crRNA21	Fig. 1d	GTTGTCATACCTATCCAAACGATAAGCTTCTAC AACAAACATTCTCAACAATAATTATAAGATTCT cctatagtgagtcgtattaatttc
Construct for crRNA22	Fig. 1d	GTTGTCATACCTATCCAAACGATAAGCTTCTAC AACAACATTCTCAACAATAATTATAAGATTCTG cctatagtgagtcgtattaatttc
Construct for crRNA23	Fig. 1d	GTTGTCATACCTATCCAAACGATAAGCTTCTAC AACACATTCTCAACAATAATTATAAGATTCTGC cctatagtgagtcgtattaatttc
Construct for crRNA24	Fig. 1d	GTTGTCATACCTATCCAAACGATAAGCTTCTAC AACCATTCTCAACAATAATTATAAGATTCTGCA cctatagtgagtcgtattaatttc
Construct for crRNA25	Fig. 1d	GTTGTCATACCTATCCAAACGATAAGCTTCTAC AACATTCTCAACAATAATTATAAGATTCTGCAG cctatagtgagtcgtattaatttc
Construct for crRNA26	Fig. 1d	GTTGTCATACCTATCCAAACGATAAGCTTCTAC AACTTCTCAACAATAATTATAAGATTCTGCAGG cctatagtgagtcgtattaatttc
Construct for crRNA27	Fig. 1d	GTTGTCATACCTATCCAAACGATAAGCTTCTAC AACTCTCAACAATAATTATAAGATTCTGCAGGC cctatagtgagtcgtattaatttc
Construct for crRNA28	Fig. 1d	GTTGTCATACCTATCCAAACGATAAGCTTCTAC AACCTCAACAATAATTATAAGATTCTGCAGGCG cctatagtgagtcgtattaatttc
Construct for crRNA29	Fig. 1d	GTTGTCATACCTATCCAAACGATAAGCTTCTAC AACTCAACAATAATTATAAGATTCTGCAGGCGG cctatagtgagtcgtattaatttc
Construct for crRNA30	Fig. 1d	GTTGTCATACCTATCCAAACGATAAGCTTCTAC AACCAACAATAATTATAAGATTCTGCAGGCGG Acctatagtgagtcgtattaatttc
Construct for crRNA31	Fig. 1d	GTTGTCATACCTATCCAAACGATAAGCTTCTAC AACAACAATAATTATAAGATTCTGCAGGCGGA Ccctatagtgagtcgtattaatttc
Construct for crRNA32	Fig. 1d	GTTGTCATACCTATCCAAACGATAAGCTTCTAC AACACAATAATTATAAGATTCTGCAGGCGGAC Ccctatagtgagtcgtattaatttc
Construct for crRNA33	Fig. 1d	GTTGTCATACCTATCCAAACGATAAGCTTCTAC AACCAATAATTATAAGATTCTGCAGGCGGACC Acctatagtgagtcgtattaatttc
Construct for crRNA34	Fig. 1d	GTTGTCATACCTATCCAAACGATAAGCTTCTAC AACAATAATTATAAGATTCTGCAGGCGGACCA Acctatagtgagtcgtattaatttc
Construct for crRNA35	Fig. 1d	GTTGTCATACCTATCCAAACGATAAGCTTCTAC AACATAATTATAAGATTCTGCAGGCGGACCAA Gcctatagtgagtcgtattaatttc
APOL1 G0G0	Figs. 3a-d, 3f, 4c-	gaaattaatacgactcactataggGCGGTGAACAGGTGGAGA GGGTTAATGAACCCAGCATCCTGGAAATGAGC

	e, 5b, 6b, EV3	AGAGGAGTCAAGCTCACGGATGTGGCCCCTGTAAGCTTCTTTCTTGTGCTGGATGTAGTCTACCTCGTGTACGAATCAAAGCACTTACATGAGGGGGCAAAGTCAGAGACAGCTGAGGAGCTGAAGAAGGTGGCTCAGGAGCTGGAGGAGAAGCTAAACATTCTCAACAATAATTATAAGATTCTGCAGGCGGACCAAGAACTGTGACCACAGGGCAGGGCAGCCACCAGGAGAGATA
APOL1 G1G1	Figs. 2c, 3a-e, 4c-e, 5b, 6b, EV2, EV3c	gaaattaatacgactcactataggGCGGTGAACAGGTGGAGAGGGTTAATGAACCCAGCATCCTGGAAATGAGCAGAGGAGTCAAGCTCACGGATGTGGCCCCTGTAGGCTTCTTTCTTGTGCTGGATGTAGTCTACCTCGTGTACGAATCAAAGCACTTACATGAGGGGGCAAAGTCAGAGACAGCTGAGGAGCTGAAGAAGGTGGCTCAGGAGCTGGAGGAGAAGCTAAACATTCTCAACAATAATTATAAGATTCTGCAGGCGGACCAAGAACTGTGACCACAGGGCAGGGCAGCCACCAGGAGAGATA
APOL1 G2G2	Figs 3a-e, 4c-e, 5b, 6b, EV2, EV3c	gaaattaatacgactcactataggAAAGCGGTGAACAGGTGGAGAGGGTTAATGAACCCAGCATCCTGGAAATGAGCAGAGGAGTCAAGCTCACGGATGTGGCCCCTGTAAGCTTCTTTCTTGTGCTGGATGTAGTCTACCTCGTGTACGAATCAAAGCACTTACATGAGGGGGCAAAGTCAGAGACAGCTGAGGAGCTGAAGAAGGTGGCTCAGGAGCTGGAGGAGAAGCTAAACATTCTCAACAATAAGATTCTGCAGGCGGACCAAGAACTGTGACCACAGGGCAGGGCAGCCACCAGGAGAGATATGC
Construct for crRNA36	Fig. 3e	GCCTGCAGAATCTTATTGTTATCTACACTTAGTAGAAATTAcctatagtgagtcgtattaatttc
Construct for crRNA37	Fig. 3e	AAGCTAAACATTCTCAACAAATCTACACTTAGTAGAAATTAcctatagtgagtcgtattaatttc
RPA primer: F1	Figs. 2a, c	gaaattaatacgactcactataggGTTAATGAACCCAGCATCCTGGAAATGAGC
RPA primer: F2	Fig. 2a	gaaattaatacgactcactataggTGAACAGGTGGAGAGGGTTAATGAACCCAG
RPA primer: F3	Fig. 2a	gaaattaatacgactcactataggTCAGCTGAAAGCGGTGAACAGGTGGAGAGG
RPA primer: R1	Fig. 2a	ATGTAAGTGCTTTGATTCGTACACGAGGTAGA
RPA primer: R2	Fig. 2a	CCCTCATGTAAGTGCTTTGATTCGTACACG
RPA primer: R3	Fig. 2a	CCTCAGCTGTCTCTGACTTTGCCCCCTCAT
RPA primer: F4	Fig. 2b	gaaattaatacgactcactataggCTGAAGAAGGTGGCTCAGGAGCTGGAGGAG
RPA primer: F5	Fig. 2b	gaaattaatacgactcactataggATCAAAGCACTTACATGAGGGGGCAAAGTC
RPA primer: F6	Fig. 2b	gaaattaatacgactcactataggAAGTCAGAGACAGCTGAGGAGCTGAAGAAG

RPA primer: R4	Fig. 2b	CTGCCCTGCCCTGTGGTCACAGTTCTTGGT
RPA primer: R5	Fig. 2b	CCCTGCCAGGCATATCTCTCCTGGTGGCTG
RPA primer: R6	Fig. 2b	AAGTTTGCATTTTGTCCTGGCCCCTGCCAG
RPA primer: F8	Fig. 2c	gaaattaatacgactcactataggGTTAATGAACCCAGCATC CTGGAAATGAG
RPA primer: F9	Fig. 2c	gaaattaatacgactcactataggTTAATGAACCCAGCATCC TGGAAATGAGC
RPA primer: R7	Fig. 2c	ACAGTTCTTGGTCCGCCTGCAGAAT
RPA primer: R8	Fig. 2c	TGCCCTGTGGTCACAGTTCTTGGTC
RPA primer: R9	Fig. 2c	CTGCCCTGTGGTCACAGTTCTTGGT
AU-Texas Red reporter molecule	Fig. 4a	5TEX615/TArArUGC/3IAbRQSp
UUUUU-Fam reporter molecule	Fig. 4a	56-FAM/rUrUrUrUrU/3IABkFQ
AAAAA-Fam reporter molecule	Fig. 4a	56-FAM/rArArArArA/3IABkFQ
AAAAA-Texas Red reporter molecule	Fig. 4a	5TEX615/rArA rArArA /3IAbRQSp
TTATT-Hex reporter molecule	Fig. 4a	5HEX/TTATT/3IABkFQ
Digoxigenin-Fam reporter	Fig. 6b	56-FAM/TTATTATT/3DigN
Biotin-Fam reporter	Fig. 6b	56-FAM/UUUUUUUUUUUUUU/3Bio
Construct for crRNA38	Fig. EV3c	GCTCACGGATGTGGCCCCTGATCTACACTTAGT AGAAATTACCTATAGTGAGTCGTATTAATTTC
Construct for crRNA39	Fig. EV3c	GCTTACAGGGGCCACATCCGATCTACACTTAGT AGAAATTACCTATAGTGAGTCGTATTAATTTC
Construct for crRNA40	Fig. EV3c	CTCACGGATGTGGCCCCTGTATCTACACTTAGT AGAAATTACCTATAGTGAGTCGTATTAATTTC
Construct for crRNA41	Fig. EV3c	CAGCACAAGAAAGAAGCTTAATCTACACTTAG TAGAAATTACCTATAGTGAGTCGTATTAATTTC
Construct for crRNA42	Fig. EV3c	CCAGCACAAGAAAGAAGCTTATCTACACTTAGT AGAAATTACCTATAGTGAGTCGTATTAATTTC
Construct for crRNA43	Fig. EV3c	TCCAGCACAAGAAAGAAGCTATCTACACTTAGT AGAAATTACCTATAGTGAGTCGTATTAATTTC
Construct for crRNA44	Fig. EV3c	AGCTCACGGATGTGGCCCCTATCTACACTTAGT AGAAATTACCTATAGTGAGTCGTATTAATTTC
Construct for crRNA45	Fig. EV3c	GCACAAGAAAGAAGCTTACAATCTACACTTAG TAGAAATTACCTATAGTGAGTCGTATTAATTTC
Construct for crRNA46	Fig. EV3d	CTCAACAATAATTATAAGATTCTGCAGGGTTTT AGTCCCCTTCGTTTTTGGGGTAGTCTAAATCcctat agtgagtcgtattaatttc
Construct for crRNA47	Fig. EV3d	TCAACAATAATTATAAGATTCTGCAGGCGTTTT AGTCCCCTTCGTTTTTGGGGTAGTCTAAATCCCT ATAGTGAGTCGTATTAATTTC
Construct for crRNA48	Fig. EV3d	CAACAATAATTATAAGATTCTGCAGGCGGTTTT AGTCCCCTTCGTTTTTGGGGTAGTCTAAATCCCT ATAGTGAGTCGTATTAATTTC

Construct for crRNA49	Fig. EV3d	AACAATAATTATAAGATTCTGCAGGCGGGTTTT AGTCCCCTTCGTTTTTGGGGTAGTCTAAATCCCT ATAGTGAGTCGTATTAATTTC
Construct for crRNA50	Fig. EV3d	ACAATAATTATAAGATTCTGCAGGCGGAGTTTT AGTCCCCTTCGTTTTTGGGGTAGTCTAAATCCCT ATAGTGAGTCGTATTAATTTC
Construct for crRNA51	Fig. EV3d	CAATAATTATAAGATTCTGCAGGCGGACGTTTT AGTCCCCTTCGTTTTTGGGGTAGTCTAAATCCCT ATAGTGAGTCGTATTAATTTC
Construct for crRNA52	Fig. EV3d	AATAATTATAAGATTCTGCAGGCGGACCGTTTT AGTCCCCTTCGTTTTTGGGGTAGTCTAAATCCCT ATAGTGAGTCGTATTAATTTC
Construct for crRNA53	Fig. EV3d	TCTCAACAATAATTATAAGATTCTGCAGGTTTT AGTCCCCTTCGTTTTTGGGGTAGTCTAAATCCCT ATAGTGAGTCGTATTAATTTC
Construct for crRNA54	Fig. EV3d	TTCTCAACAATAATTATAAGATTCTGCAGTTTT AGTCCCCTTCGTTTTTGGGGTAGTCTAAATCCCT ATAGTGAGTCGTATTAATTTC
Construct for crRNA55	Fig. EV3d	ATTCTCAACAATAATTATAAGATTCTGCGTTTT AGTCCCCTTCGTTTTTGGGGTAGTCTAAATCCCT ATAGTGAGTCGTATTAATTTC
Construct for crRNA56	Fig. EV3d	CATTCTCAACAATAATTATAAGATTCTGGTTTT AGTCCCCTTCGTTTTTGGGGTAGTCTAAATCCCT ATAGTGAGTCGTATTAATTTC
Construct for crRNA57	Fig. EV3d	ACATTCTCAACAATAATTATAAGATTCTGTTTT AGTCCCCTTCGTTTTTGGGGTAGTCTAAATCCCT ATAGTGAGTCGTATTAATTTC
Construct for crRNA58	Fig. EV3d	AACATTCTCAACAATAATTATAAGATTCGTTTT AGTCCCCTTCGTTTTTGGGGTAGTCTAAATCCCT ATAGTGAGTCGTATTAATTTC
Construct for crRNA59	Fig. EV3d	AAACATTCTCAACAATAATTATAAGATTGTTTT AGTCCCCTTCGTTTTTGGGGTAGTCTAAATCCCT ATAGTGAGTCGTATTAATTTC
Construct for crRNA60	Fig. EV3d	AGCTAAACATTCTCAACAATAATTATAAGTTTT AGTCCCCTTCGTTTTTGGGGTAGTCTAAATCcctat agtgagtcgtattaatttc
Construct for crRNA61	Fig. EV3d	AGAAGCTAAACATTCTCAACAATAATTAGTTTT AGTCCCCTTCGTTTTTGGGGTAGTCTAAATCCCT ATAGTGAGTCGTATTAATTTC
Construct for crRNA62	Fig. EV3d	TAAACATTCTCAACAATAATTATAAGATGTTTT AGTCCCCTTCGTTTTTGGGGTAGTCTAAATCCCT ATAGTGAGTCGTATTAATTTC
Construct for crRNA63	Fig. EV3d	CTAAACATTCTCAACAATAATTATAAGAGTTTT AGTCCCCTTCGTTTTTGGGGTAGTCTAAATCCCT ATAGTGAGTCGTATTAATTTC

Construct for crRNA64	Fig. EV3d	GCTAAACATTCTCAACAATAATTATAAGGTTTTAGTCCCCTTCGTTTTTGGGGTAGTCTAAATCCCTATAGTGAGTCGTATTAATTTC
Construct for crRNA65	Fig. EV3d	AAGCTAAACATTCTCAACAATAATTATAGTTTTAGTCCCCTTCGTTTTTGGGGTAGTCTAAATCCCTATAGTGAGTCGTATTAATTTC
Construct for crRNA66	Fig. EV3d	GAAGCTAAACATTCTCAACAATAATTATGTTTTAGTCCCCTTCGTTTTTGGGGTAGTCTAAATCCCTATAGTGAGTCGTATTAATTTC
PCR reverse primer for sequencing		CTGCCAGGCATATCTCTCCT
PCR forward primer for sequencing		gaaattaatacgactcactataggACCAACTCACACGAGGCATT

Appendix Table S2: P values.

Description	Figure	P value
crRNA 1	Fig. 1b	0.0007
crRNA 2	Fig. 1b	<0.0001
crRNA 3	Fig. 1b	<0.0001
crRNA 4	Fig. 1b	0.0004
crRNA 5	Fig. 1b	0.0024
crRNA 6	Fig. 1b	0.0063
crRNA 7	Fig. 1b	0.0006
crRNA 8	Fig. 1b	0.007
crRNA 9	Fig. 1b	<0.0001
crRNA 10	Fig. 1d	0.3263
crRNA 11	Fig. 1d	0.0004
crRNA 12	Fig. 1d	<0.0001
crRNA 13	Fig. 1d	0.0004
crRNA 14	Fig. 1d	0.0002
crRNA 15	Fig. 1d	0.0002
crRNA 16	Fig. 1d	<0.0001
crRNA 17	Fig. 1d	<0.0001
crRNA 18	Fig. 1d	<0.0001
crRNA 19	Fig. 1d	<0.0001
crRNA 20	Fig. 1d	<0.0001
crRNA 21	Fig. 1d	<0.0001
crRNA 22	Fig. 1d	<0.0001
crRNA 23	Fig. 1d	0.0001
crRNA 24	Fig. 1d	0.0027
crRNA 25	Fig. 1d	<0.0001
crRNA 26	Fig. 1d	<0.0001
crRNA 27	Fig. 1d	<0.0001

crRNA 28	Fig. 1d	0.0002
crRNA 29	Fig. 1d	<0.0001
crRNA 30	Fig. 1d	0.0002
crRNA 31	Fig. 1d	<0.0001
crRNA 32	Fig. 1d	0.0008
crRNA 33	Fig. 1d	<0.0001
crRNA 34	Fig. 1d	<0.0001
crRNA 35	Fig. 1d	0.0017
F2R1 (1 pM)	Fig. 2a	0.9980
F2R1 (10 fM)	Fig. 2a	0.0392
F3R1 (1 pM)	Fig. 2a	0.0376
F3R1 (10 fM)	Fig. 2a	0.1417
F1R2 (1 pM)	Fig. 2a	<0.0001
F1R2 (10 fM)	Fig. 2a	<0.0001
F2R2 (1 pM)	Fig. 2a	<0.0001
F2R2 (10 fM)	Fig. 2a	<0.0001
F3R2 (1 pM)	Fig. 2a	<0.0001
F3R2 (10 fM)	Fig. 2a	<0.0001
F1R3 (1 pM)	Fig. 2a	<0.0001
F1R3 (10 fM)	Fig. 2a	<0.0001
F2R3 (1 pM)	Fig. 2a	<0.0001
F2R3 (10 fM)	Fig. 2a	<0.0001
F3R3 (1 pM)	Fig. 2a	<0.0001
F3R3 (10 fM)	Fig. 2a	<0.0001
F4R4 (1 pM)	Fig. 2b	<0.0001
F4R4 (10 fM)	Fig. 2b	<0.0001
F5R4 (1 pM)	Fig. 2b	<0.0001
F5R4 (10 fM)	Fig. 2b	<0.0001
F6R4 (1 pM)	Fig. 2b	<0.0001
F6R4 (10 fM)	Fig. 2b	<0.0001

F4R5 (1 pM)	Fig. 2b	0.9970
F4R5 (10 fM)	Fig. 2b	<0.0001
F6R5 (1 pM)	Fig. 2b	0.5757
F6R5 (10 fM)	Fig. 2b	<0.0001
F4R6 (1 pM)	Fig. 2b	0.9220
F4R6 (10 fM)	Fig. 2b	<0.0001
F5R6 (1 pM)	Fig. 2b	0.9690
F5R6 (10 fM)	Fig. 2b	<0.0001
F6R6 (1 pM)	Fig. 2b	0.2113
F6R6 (10 fM)	Fig. 2b	<0.0001
F1R7 (1 pM)	Fig. 2c	0.9997
F1R7 (10 fM)	Fig. 2c	<0.0001
F8R7 (1 pM)	Fig. 2c	>0.9999
F8R7 (10 fM)	Fig. 2c	<0.0001
F7R8 (1 pM)	Fig. 2c	<0.0001
F7R8 (10 fM)	Fig. 2c	<0.0001
F8R8 (1 pM)	Fig. 2c	<0.0001
F8R8 (10 fM)	Fig. 2c	<0.0001
F9R8 (1 pM)	Fig. 2c	0.9971
F9R8 (10 fM)	Fig. 2c	<0.0001
F7R9 (1 pM)	Fig. 2c	<0.0001
F7R9 (10 fM)	Fig. 2c	<0.0001
F8R9 (1 pM)	Fig. 2c	<0.0001
F8R9 (10 fM)	Fig. 2c	<0.0001
F9R9 (1 pM)	Fig. 2c	0.0001
F9R9 (10 fM)	Fig. 2c	<0.0001
crRNA 36	Fig. 3f	<0.0001
crRNA 37	Fig. 3f	<0.0001
crRNA 38	Fig. EV3b	0.8309
crRNA 39	Fig. EV3b	0.204

crRNA 40	Fig. EV3b	0.0448
crRNA 41	Fig. EV3b	<0.0001
crRNA 42	Fig. EV3b	<0.0001
crRNA 43	Fig. EV3b	0.5172
crRNA 44	Fig. EV3b	0.9474
crRNA 45	Fig. EV3b	<0.0001
crRNA 46	Fig. EV3d	0.0054
crRNA 47	Fig. EV3d	0.0001
crRNA 48	Fig. EV3d	0.0106
crRNA 49	Fig. EV3d	<0.0001
crRNA 50	Fig. EV3d	<0.0001
crRNA 51	Fig. EV3d	0.0004
crRNA 52	Fig. EV3d	0.1664
crRNA 53	Fig. EV3d	<0.0001
crRNA 54	Fig. EV3d	0.0042
crRNA 55	Fig. EV3d	<0.0001
crRNA 56	Fig. EV3d	<0.0001
crRNA 57	Fig. EV3d	0.0033
crRNA 58	Fig. EV3d	<0.0001
crRNA 59	Fig. EV3d	0.0011
crRNA 60	Fig. EV3d	0.0008
crRNA 61	Fig. EV3d	0.0001
crRNA 62	Fig. EV3d	<0.0001
crRNA 63	Fig. EV3d	<0.0001
crRNA 64	Fig. EV3d	<0.0001
crRNA 65	Fig. EV3d	0.0017
crRNA 66	Fig. EV3d	0.0011

Appendix Table S3: APOL1 Genotyping Cost Analysis.

**APOL1 genotyping methods**	**PCR followed by Sanger sequencing**	**Clinical diagnostics service**	**CRISPR (lateral-flow)**	**CRISPR (fluorescence)**
**Reference**	New England Biolabs[a]. Eurofins Genomics[b].	Wake Forest University School of Medicine[c]. Mass General Brigham Laboratory for Molecular Medicine[d].	This paper.	This paper.
**Time to result**	~ 1 day	7 days - 21 days	~ 2.5 hours	~ 2.5 hours
**Service cost (€)**	~ 4 (sequencing)	~ 360 - 550	NA	NA
**Equipment cost (€)**	>10,000 (cycler)	NA	2.90/ stick[e] 250 (heatblock*)	>10,000 (plate reader)
**Reagent costs (€)**				
PCR reagents	1.101	NA	NA	NA
PCR primers	0.002	NA	NA	NA
RPA reagents	NA	NA	0.45	0.45
RPA primers	NA	NA	0.02	0.02
Cas enzymes	NA	NA	1.24	0.86
crRNAs	NA	NA	0.04	0.01
Reporter molecules	NA	NA	0.06	0.64
Other (buffer/ rnase inhibitor/ rNTPs/ T7 polymerase)	NA	NA	1.22	0.64
Total reagent costs (€)	1.1	NA	3.02	2.62
**Hands-on time**	<1 hr	NA	<1 hr	<1 hr
**POC compatibility**				
Isothermal incubation	No	No	Yes	Yes
Minimal equipment	No	No	Yes	No
Visual output	No	No	Yes	No

a. New England Biolabs Q5 High Fidelity DNA Polymerase (https://www.neb-online.de/art/M0491).
b. Eurofins Genomics Mix2Seq Kits (https://eurofinsgenomics.eu/de/custom-dna-sequencing/eurofins-services/mix2seq-kits/).
c. *Wake Forest University School of Medicine; APOL1 Genetic Test* (https://school.wakehealth.edu/departments/internal-medicine/nephrology/nephrology-research/apol1-genetic-test).
d. *Mass General Brigham Laboratory for Molecular Medicine* (https://www.massgeneralbrigham.org/en/research-and-innovation/centers-and-programs/personalized-medicine/molecular-medicine/tests/pricing-turnaround-time-cpt-codes).
e. Milenia Biotec HybriDetect 2T (https://www.milenia-biotec.com/en/product/hybridetect-2t/).
* CRISPR-based diagnostics assays have been reported which could be completed without heatblocks and simply used body heat as a heat source: Arizti-Sanz *et al.* Simplified Cas13-based assays for the fast identification of SARS-CoV-2 and its variants. *Nat. Biomed. Eng* **6**, 932–943 (2022).
