# Supplementary figures and images for: CRISPR-enabled point-of-care genotyping for APOL1 genetic risk assessment
Source: EMBO Mol Med. 2024 Sep 13;16(10):18. doi: 10.1038/s44321-024-00126-x (PMC11473833; doi:10.1038/s44321-024-00126-x)

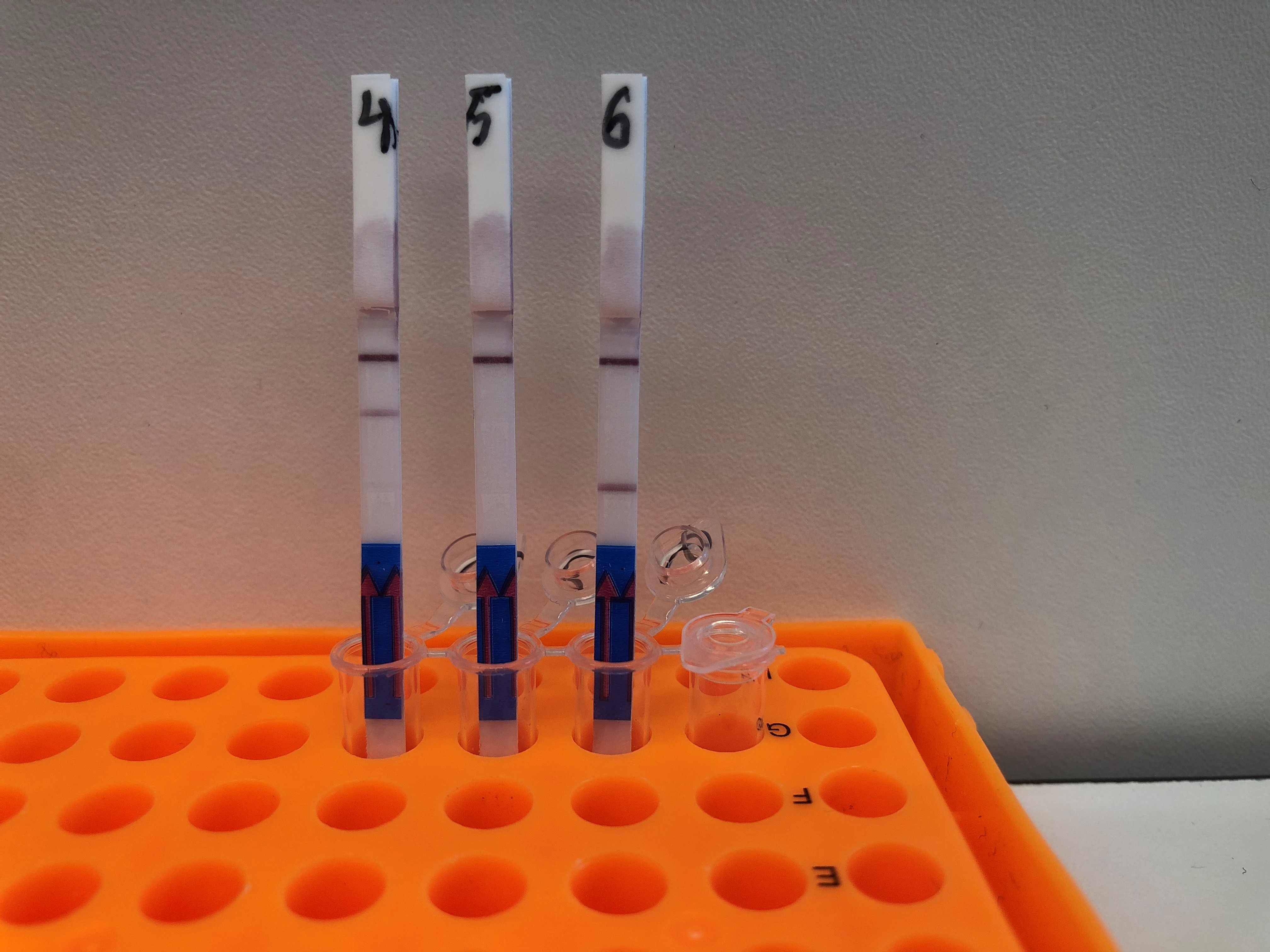

Supplement: Supplementary file 9 — Source data Fig. 6 [file 44321_2024_126_MOESM9_ESM.zip › EMM-2024-19522_SourceDataForFigure6/EMM-2024-19522_SourceDataForFigure6B_images_gDNA/G1 assay test 1.jpg]

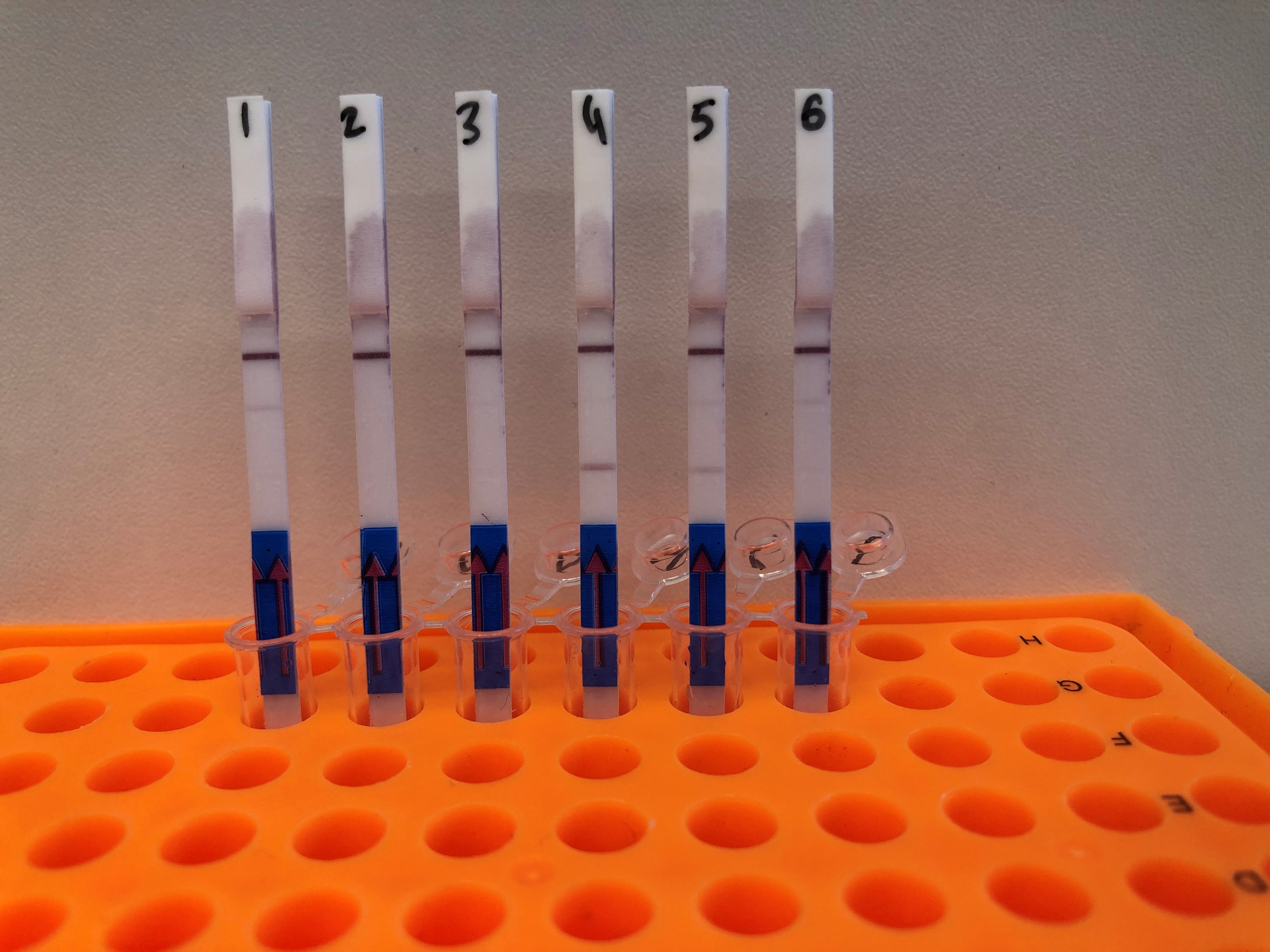

Supplement: Supplementary file 9 — Source data Fig. 6 [file 44321_2024_126_MOESM9_ESM.zip › EMM-2024-19522_SourceDataForFigure6/EMM-2024-19522_SourceDataForFigure6B_images_gDNA/G1 assay test 2.jpg]

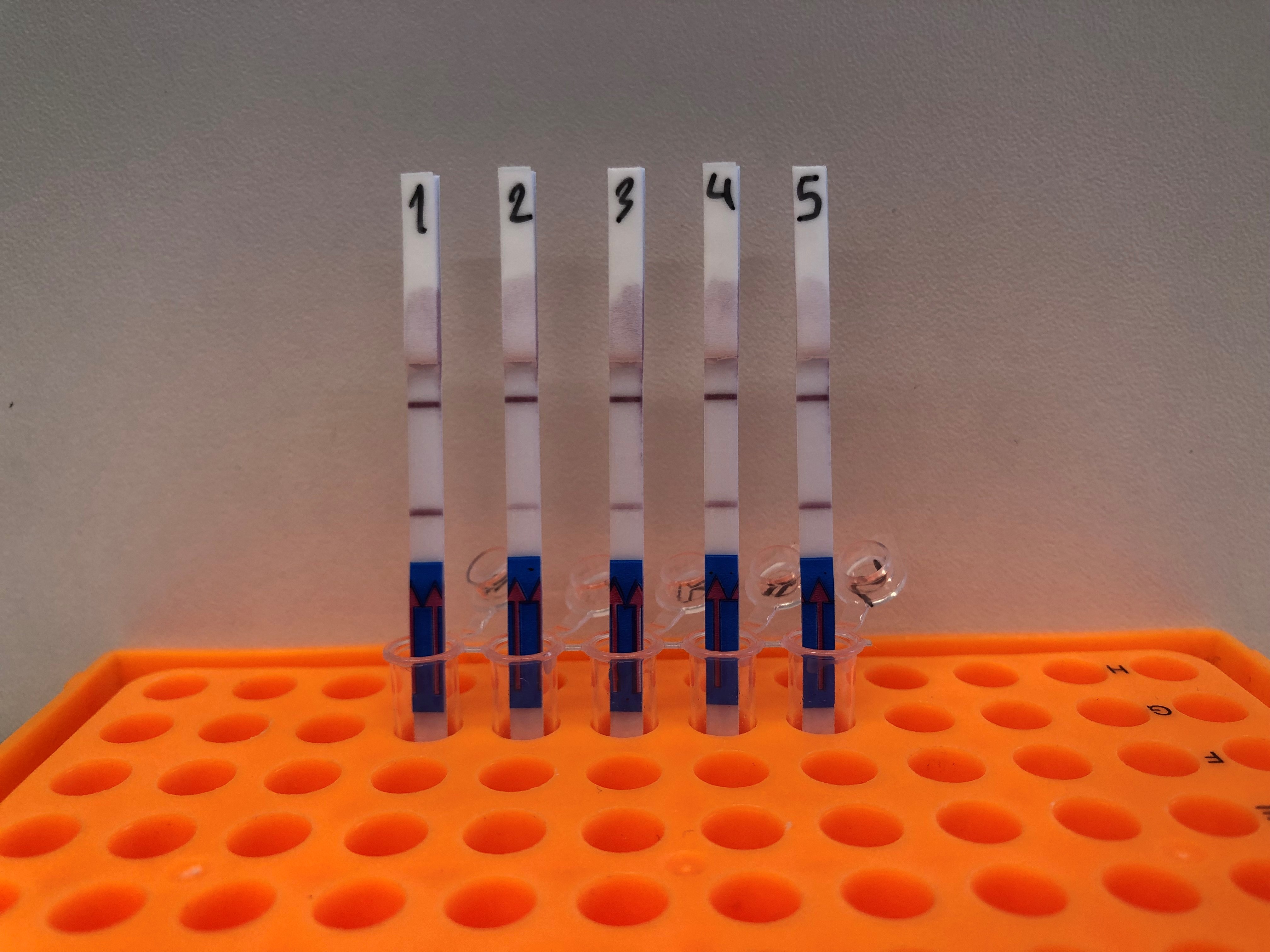

Supplement: Supplementary file 9 — Source data Fig. 6 [file 44321_2024_126_MOESM9_ESM.zip › EMM-2024-19522_SourceDataForFigure6/EMM-2024-19522_SourceDataForFigure6B_images_gDNA/G1 assay test 3.jpg]

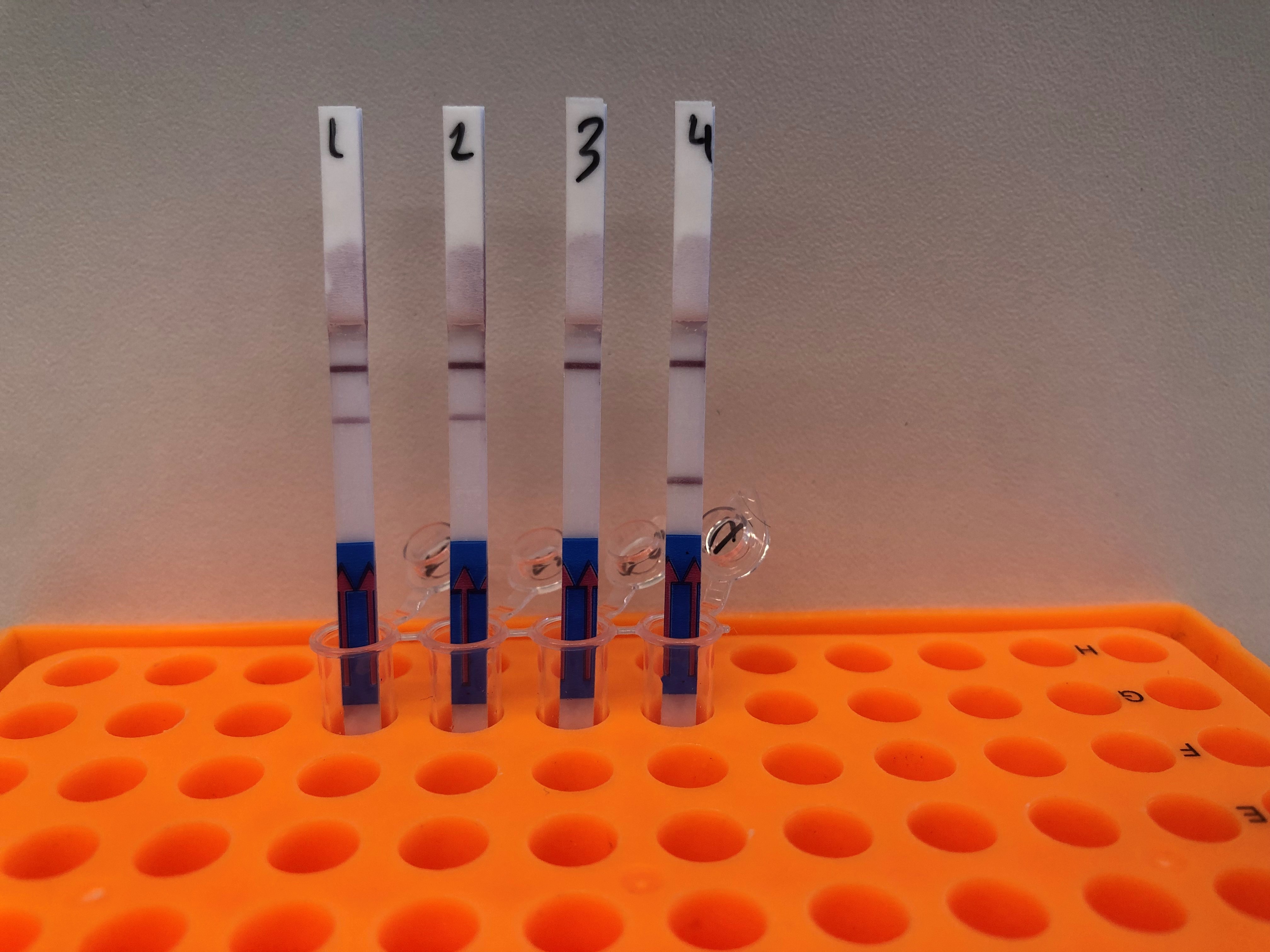

Supplement: Supplementary file 9 — Source data Fig. 6 [file 44321_2024_126_MOESM9_ESM.zip › EMM-2024-19522_SourceDataForFigure6/EMM-2024-19522_SourceDataForFigure6B_images_gDNA/G1 assay test 4.jpg]

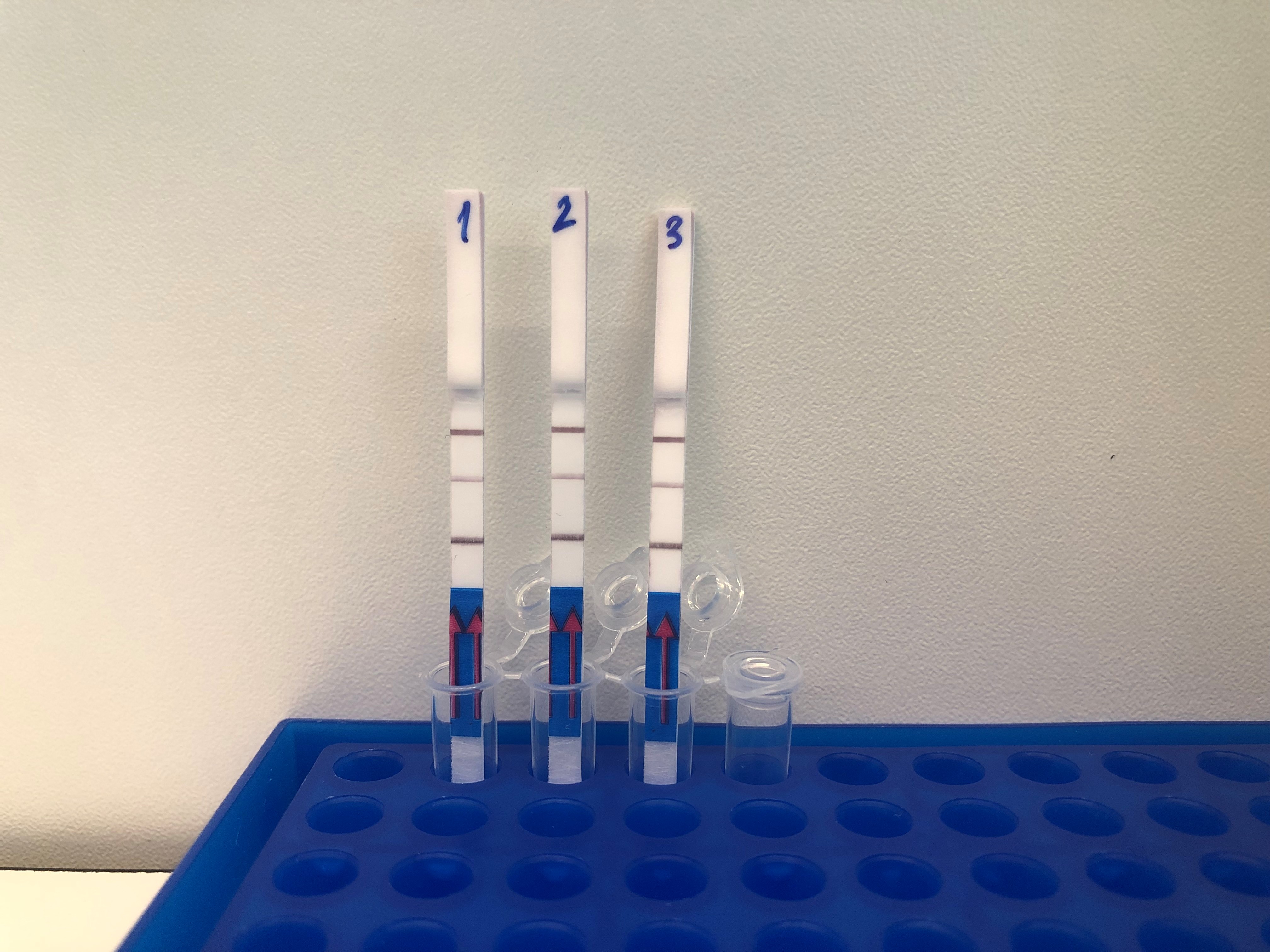

Supplement: Supplementary file 9 — Source data Fig. 6 [file 44321_2024_126_MOESM9_ESM.zip › EMM-2024-19522_SourceDataForFigure6/EMM-2024-19522_SourceDataForFigure6B_images_gDNA/G1 assay test 5.jpg]

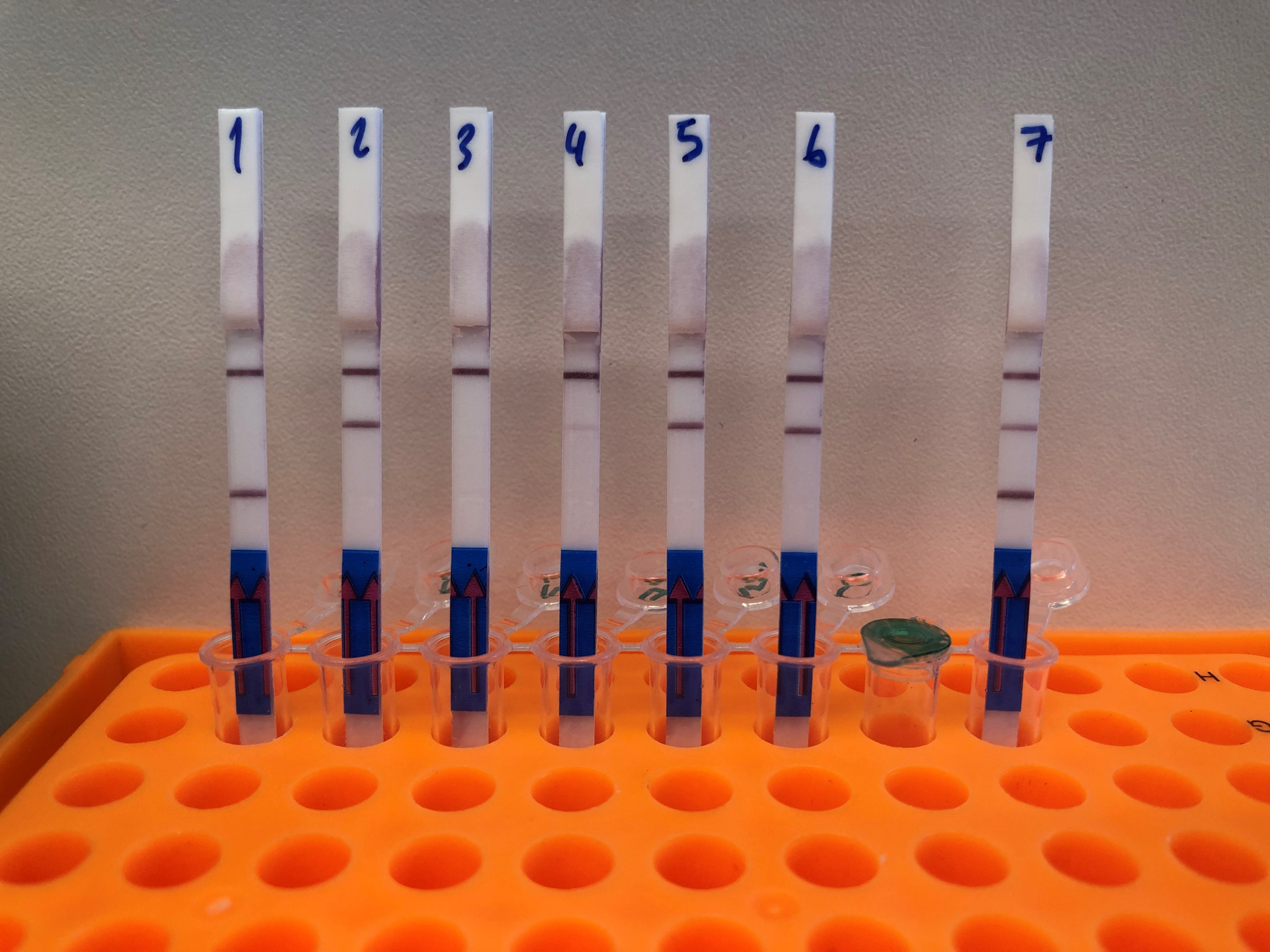

Supplement: Supplementary file 9 — Source data Fig. 6 [file 44321_2024_126_MOESM9_ESM.zip › EMM-2024-19522_SourceDataForFigure6/EMM-2024-19522_SourceDataForFigure6B_images_gDNA/G2 aassay test 2.jpg]

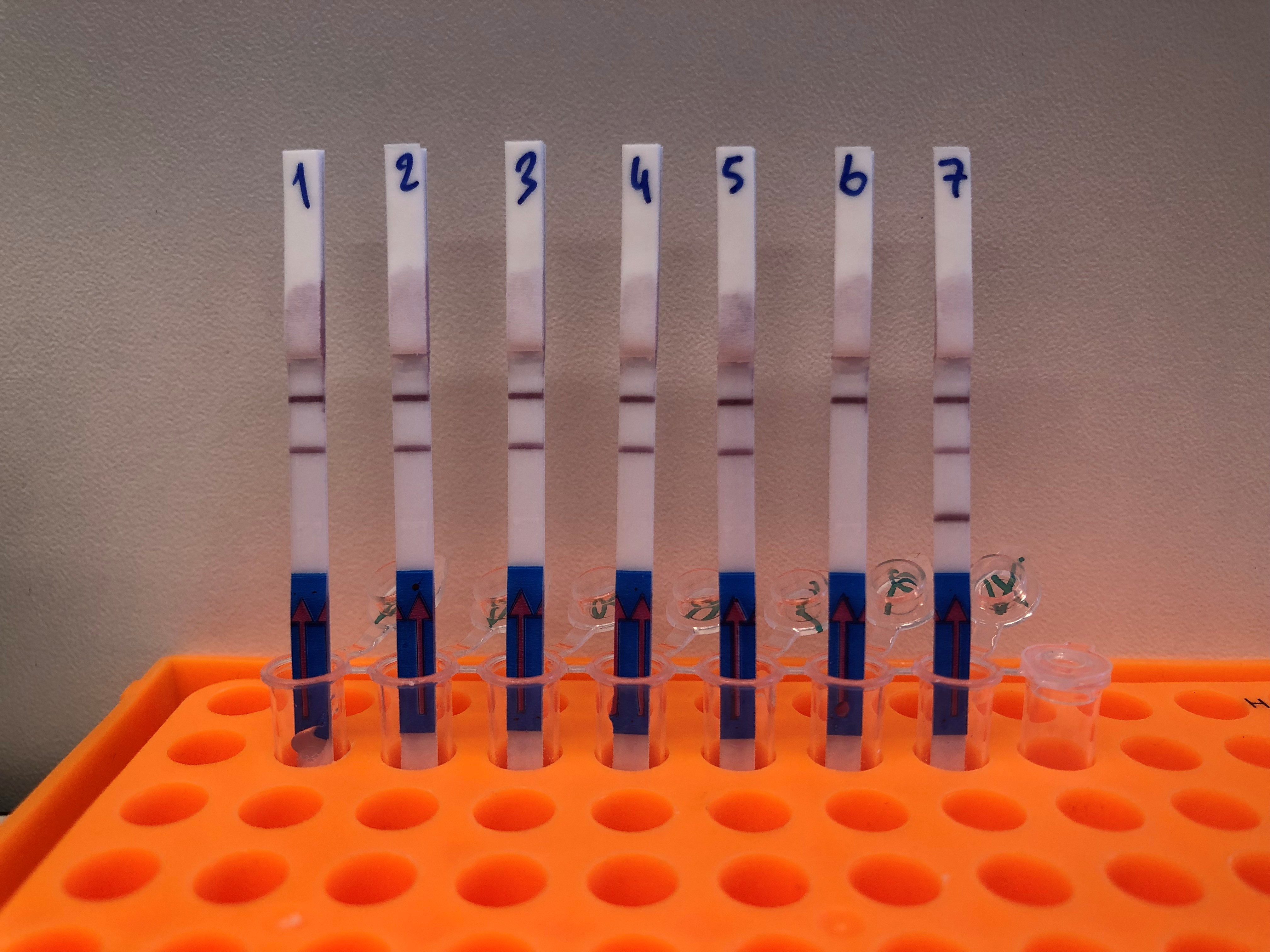

Supplement: Supplementary file 9 — Source data Fig. 6 [file 44321_2024_126_MOESM9_ESM.zip › EMM-2024-19522_SourceDataForFigure6/EMM-2024-19522_SourceDataForFigure6B_images_gDNA/G2 assay test 1.jpg]

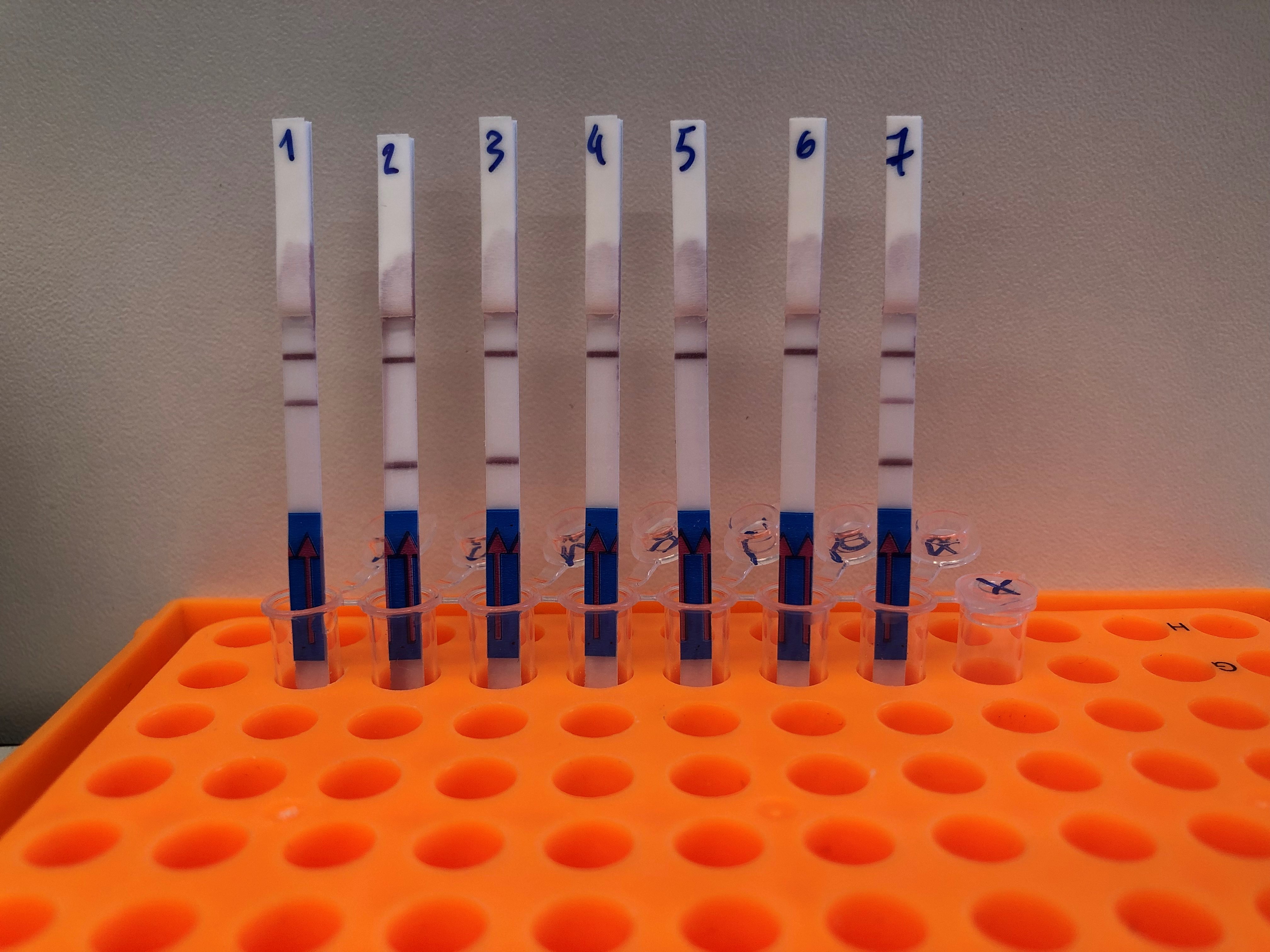

Supplement: Supplementary file 9 — Source data Fig. 6 [file 44321_2024_126_MOESM9_ESM.zip › EMM-2024-19522_SourceDataForFigure6/EMM-2024-19522_SourceDataForFigure6B_images_gDNA/G2 assay test 3.jpg]

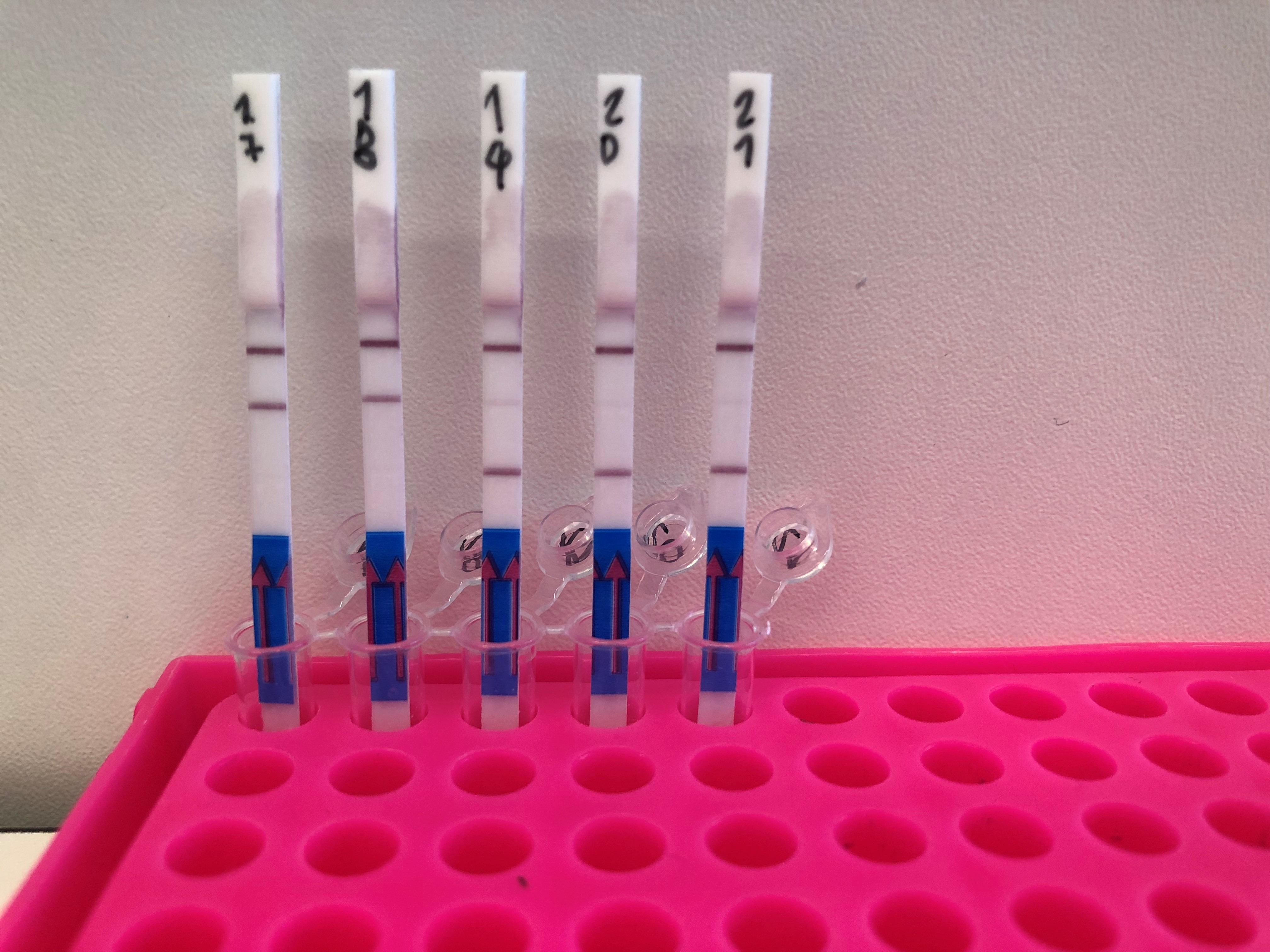

Supplement: Supplementary file 9 — Source data Fig. 6 [file 44321_2024_126_MOESM9_ESM.zip › EMM-2024-19522_SourceDataForFigure6/EMM-2024-19522_SourceDataForFigure6B_images_synthetic/17-21.jpg]

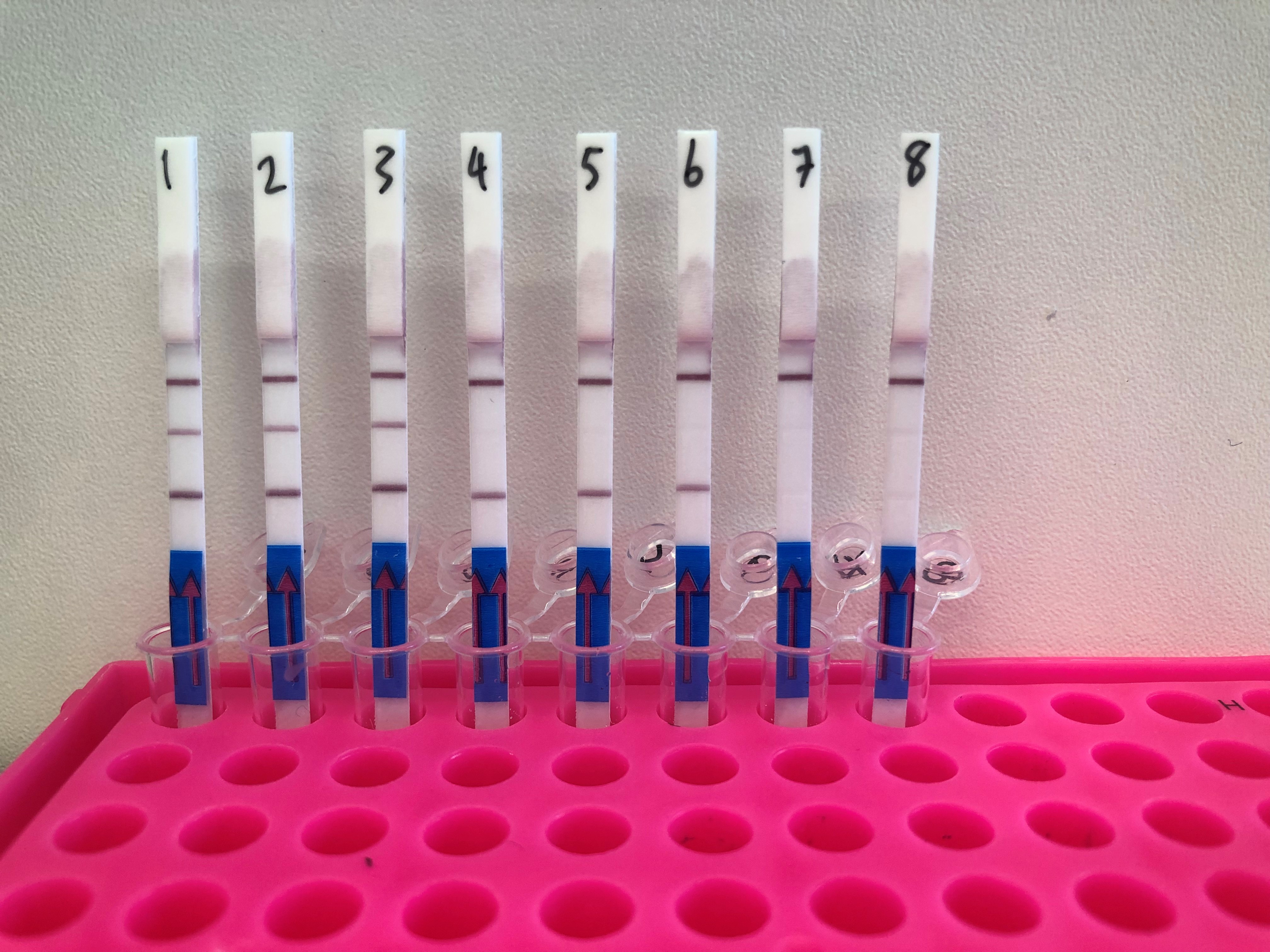

Supplement: Supplementary file 9 — Source data Fig. 6 [file 44321_2024_126_MOESM9_ESM.zip › EMM-2024-19522_SourceDataForFigure6/EMM-2024-19522_SourceDataForFigure6B_images_synthetic/1-8.jpg]

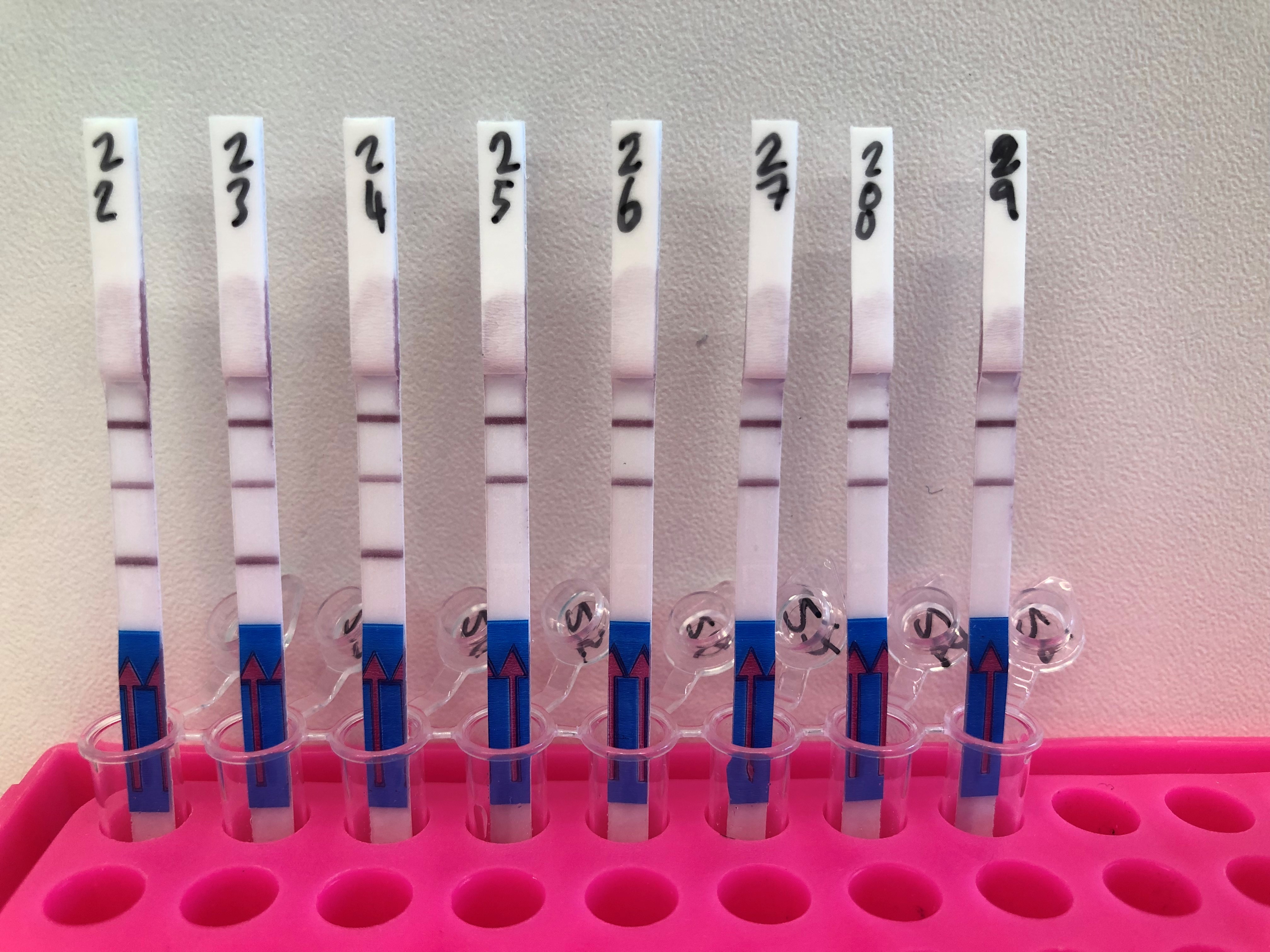

Supplement: Supplementary file 9 — Source data Fig. 6 [file 44321_2024_126_MOESM9_ESM.zip › EMM-2024-19522_SourceDataForFigure6/EMM-2024-19522_SourceDataForFigure6B_images_synthetic/22-29.jpg]

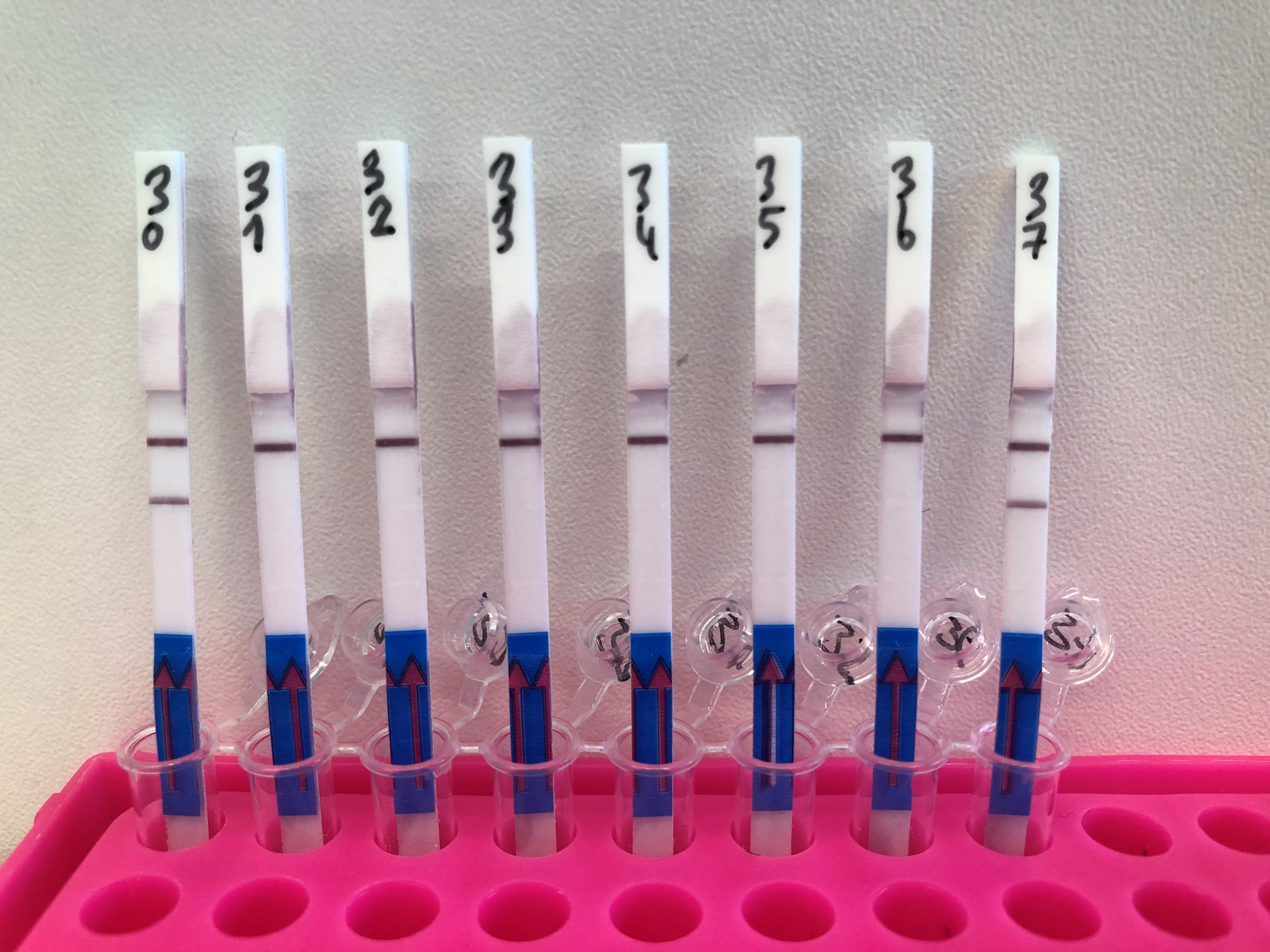

Supplement: Supplementary file 9 — Source data Fig. 6 [file 44321_2024_126_MOESM9_ESM.zip › EMM-2024-19522_SourceDataForFigure6/EMM-2024-19522_SourceDataForFigure6B_images_synthetic/30-37.jpg]

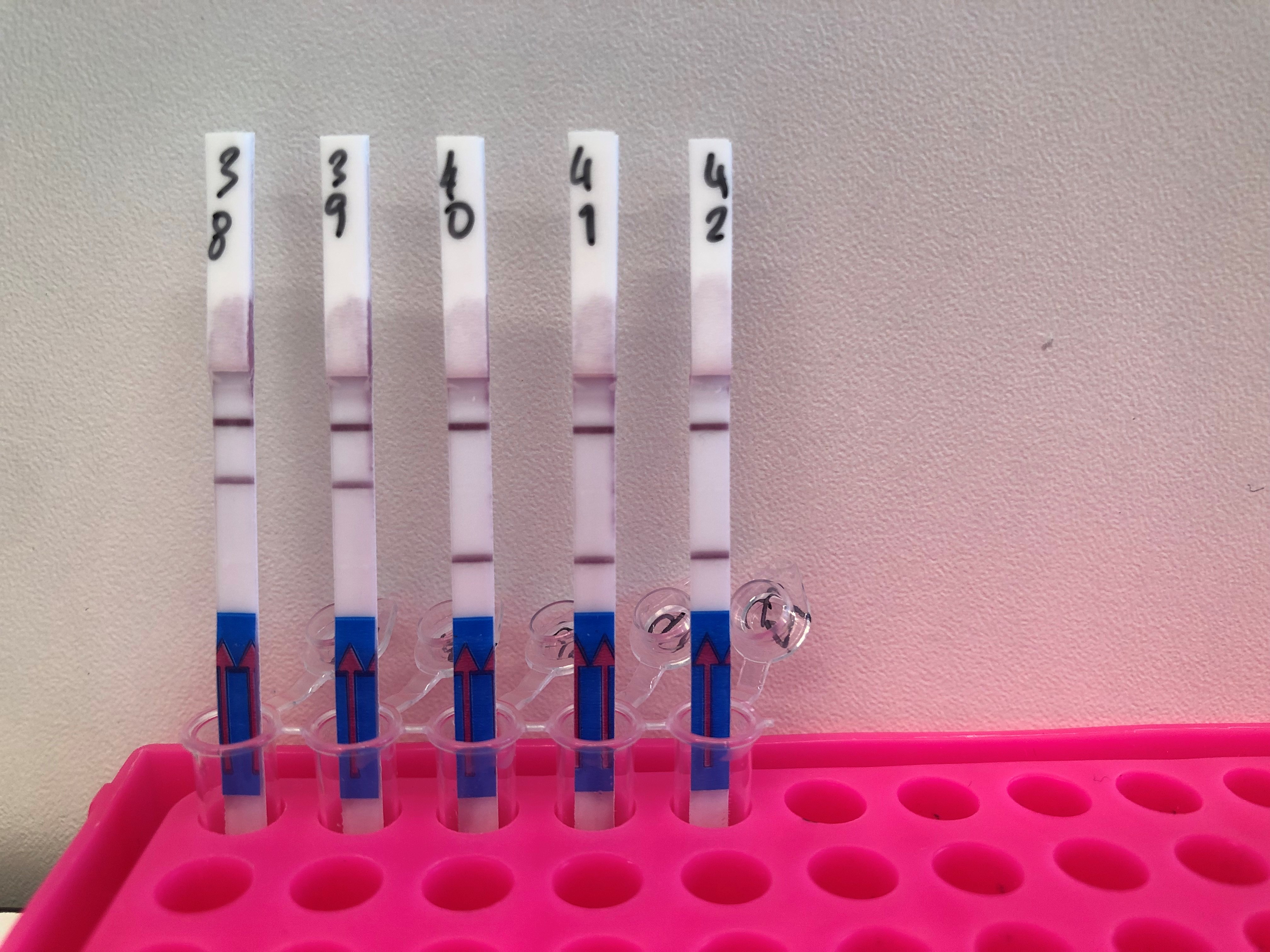

Supplement: Supplementary file 9 — Source data Fig. 6 [file 44321_2024_126_MOESM9_ESM.zip › EMM-2024-19522_SourceDataForFigure6/EMM-2024-19522_SourceDataForFigure6B_images_synthetic/38-42.jpg]

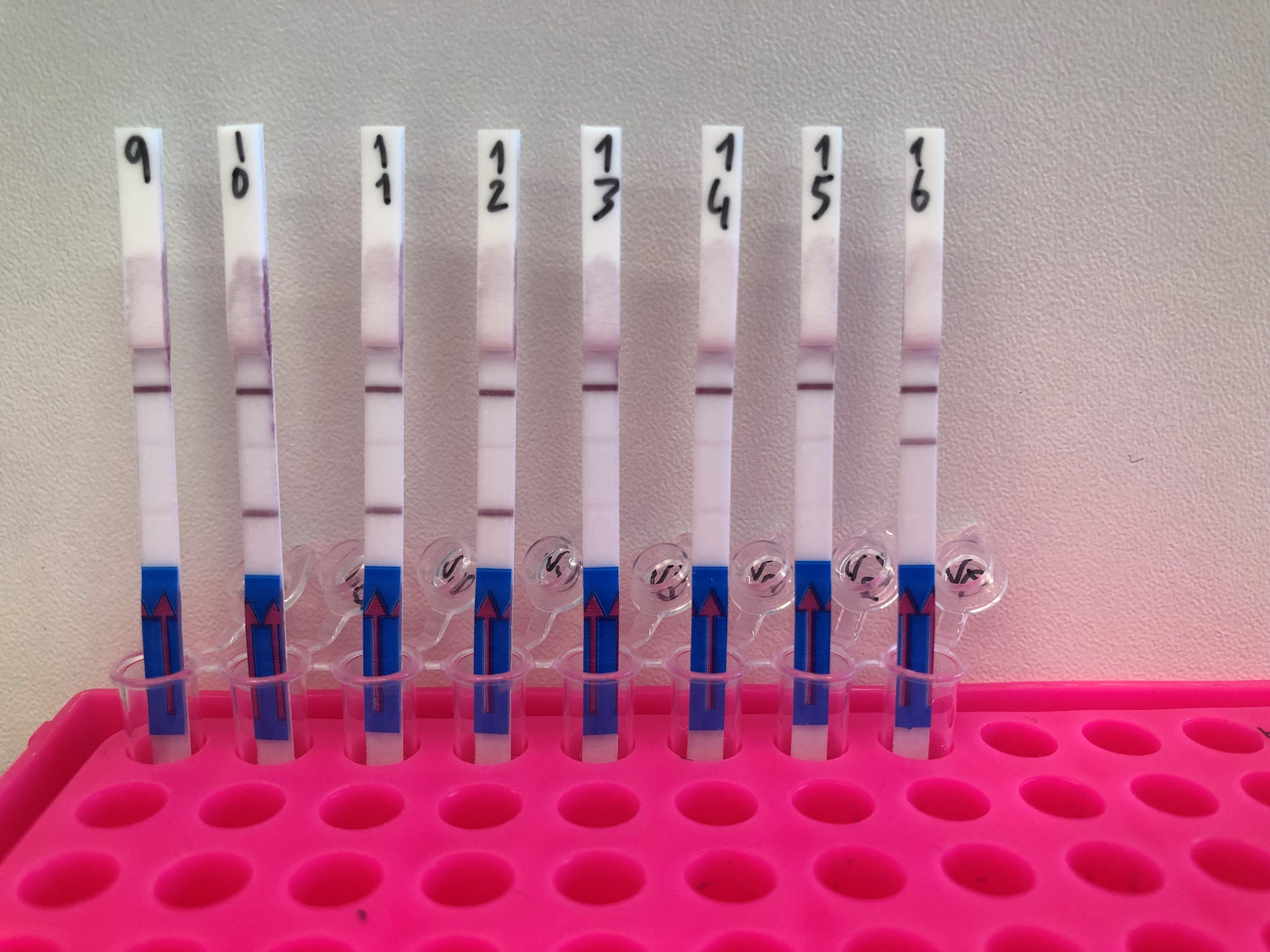

Supplement: Supplementary file 9 — Source data Fig. 6 [file 44321_2024_126_MOESM9_ESM.zip › EMM-2024-19522_SourceDataForFigure6/EMM-2024-19522_SourceDataForFigure6B_images_synthetic/9-16.jpg]
