# Supplementary material for: CRISPR-enabled point-of-care genotyping for APOL1 genetic risk assessment
Source: EMBO Mol Med. 2024 Sep 13;16(10):18. doi: 10.1038/s44321-024-00126-x (PMC11473833; doi:10.1038/s44321-024-00126-x)
Supplement: Supplementary file 10 — Expanded View Figures [file 44321_2024_126_MOESM10_ESM.pdf]

Expanded View Figures

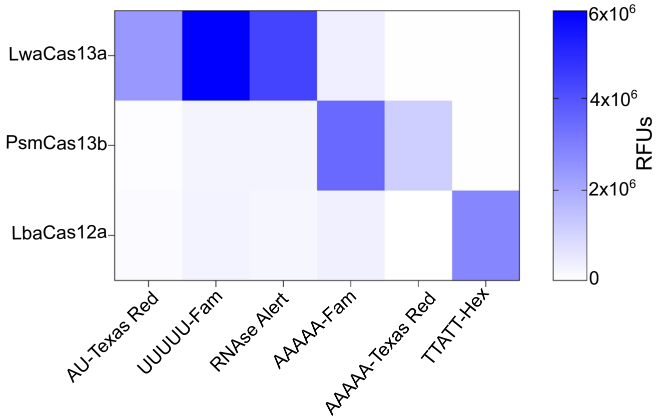

Figure EV1. Orthogonality of Cas enzyme cleavage preference.

CRISPR assay sensing synthetic RNA containing the target allele at an overall concentration of 15 nM (LwaCas13a; PsmCas13b) or DNA at an overall concentration of 300 pM (LbaCas12a). RFUs are shown at 3 h and heatmaps visualize the mean of 3 technical replicates. Source data are available online for this figure.

 © The Author(s)

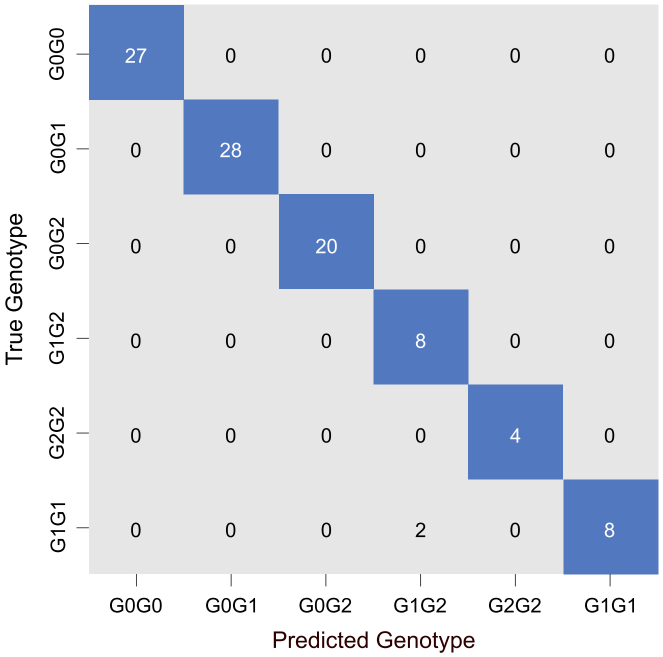

Figure EV2. CRISPR-based *APOL1* genotyping by genotype scores.

Confusion matrix summarizing accuracy of CRISPR-based (predicted) genotyping as calculated by the genotype score method for six *APOL1* genotypes as compared to Sanger Sequencing determined (true) genotypes for US and German cohort as shown in Fig. 5A. Numbers indicate patients and boxes shaded blue indicate a true positive result. Source data are available online for this figure.

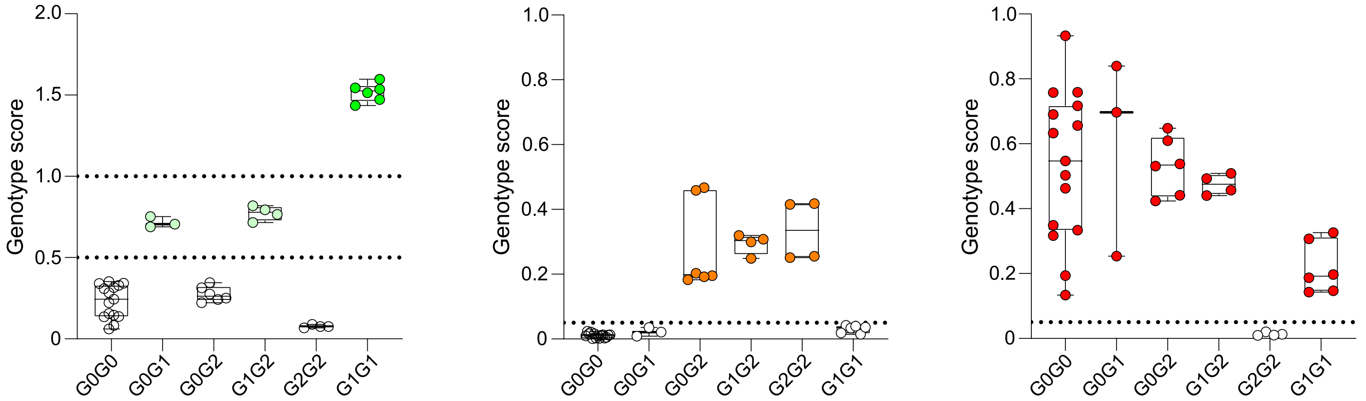

Figure EV3. *APOL1* genotyping of independent Brazilian cohort.

Box plots show genotype scores of patient samples from the Brazilian cohort. The data for LwaCas13a, LbaCas12a, and PsmCas13b are shown on the left, middle, and right, respectively. Each data point represents an individual patient. Dashed lines indicate genotype score thresholds. Within each box-whisker plot, whiskers extend from the minimum to the maximum values; boxes extend from the 25th to the 75th percentiles; the median value is annotated by a horizontal line through the box. Source data are available online for this figure.

 © The Author(s)

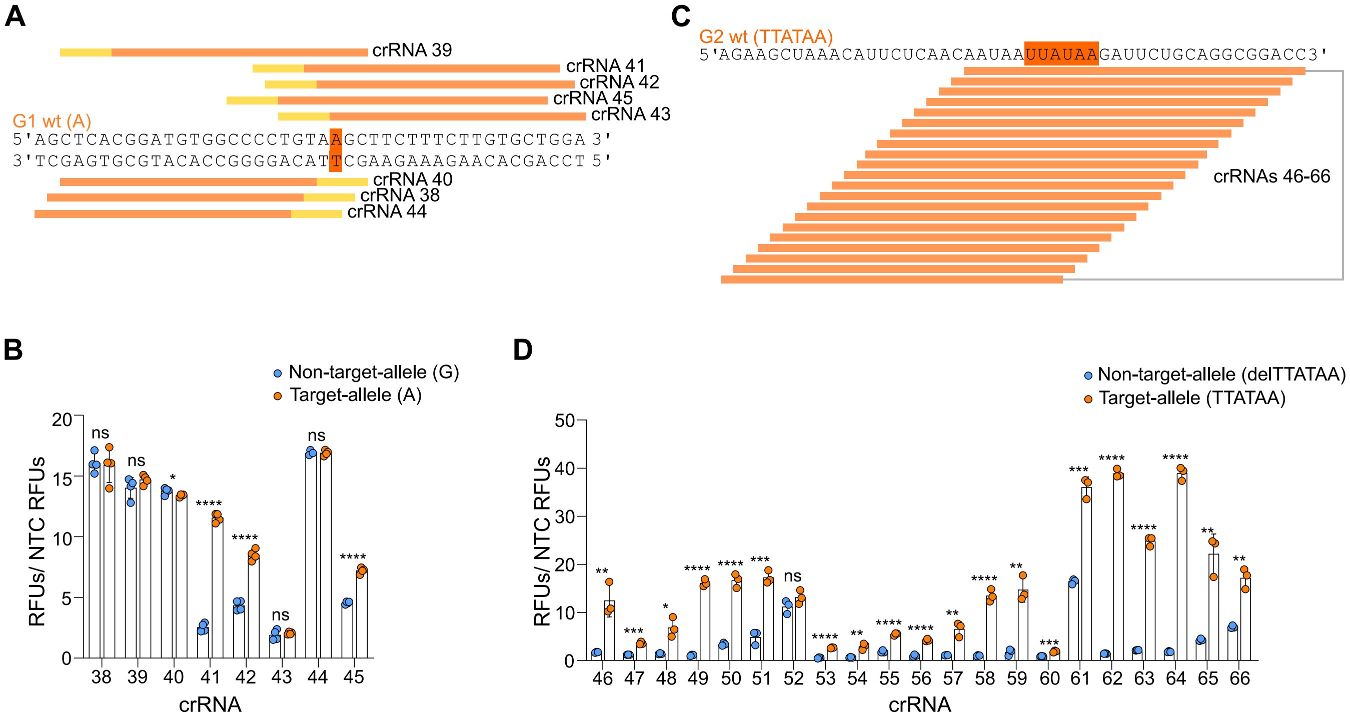

Figure EV4. crRNA screening for lateral-flow-based *APOL1* genotyping.

(**A**) Alignment of target sequences and LbaCas12a G1 wt sensing crRNAs. The spacers of the crRNAs are shown in orange, and the corresponding PAMs are shown in yellow. G1 wt allele highlighted in dark orange. (**B**) Screen for G1 wt sensing LbaCas12a crRNAs detecting synthetic DNA containing target- (wt; orange) and non-target- (mut; blue) alleles. DNA was detected at an overall concentration of 100 fM. RPA was performed in duplicates for each sample and each RPA product was measured twice with the CRISPR assay. Each data point represents an individual reaction. For each reaction, relative RFUs are shown at 2 h divided by the mean NTC RFU value. (**C**) Alignment of target sequence and LwaCas13a G2 wt sensing crRNAs. Dark orange highlights the position of the 6 bp G2 wt sequence and crRNA spacers are shown in light orange. (**D**) Screen for G2 wt sensing LwaCas13a crRNAs detecting synthetic RNA containing target- (orange) and non-target- (blue) alleles. RNA detected at an overall concentration of 15 nM, each data point represents an individual reaction. RFUs are shown at 3 h divided by the mean RFU value of the non-template control. (**B**–**D**) Error bars: s.d. Statistical significance assessed with unpaired *t* test; $P > 0.05$= not significant (ns), $^{*}P \le 0.05$, $^{**}P \le 0.01$, $^{***}P \le 0.001$ and $^{****}P \le 0.0001$. For *P* values see: Appendix Table S2. Source data are available online for this figure.

© The Author(s)
